# Assessing global dietary habits: a comparison of national estimates from the FAO and the Global Dietary Database^1^,^2^,^3^,^4^

**DOI:** 10.3945/ajcn.114.087403

**Published:** 2015-03-18

**Authors:** Liana C Del Gobbo, Shahab Khatibzadeh, Fumiaki Imamura, Renata Micha, Peilin Shi, Matthew Smith, Samuel S Myers, Dariush Mozaffarian

**Affiliations:** 1 From the Departments of Nutrition (LCDG), Epidemiology (RM, PS, and DM), and Environmental Health (MS and SSM), Harvard School of Public Health, Boston, MA; the Medical Research Council Epidemiology Unit, Institute of Metabolic Science, University of Cambridge School of Clinical Medicine, Cambridge Biomedical Campus, Cambridge, United Kingdom (FI); and the Department of Food Science and Human Nutrition, Agricultural University of Athens, Athens, Greece (RM).

**Keywords:** dietary intakes, food balance sheets, global diet, FAO, nutrition, food availability

## Abstract

**Background:** Accurate data on dietary habits are crucial for understanding impacts on disease and informing policy priorities. Nation-specific food balance sheets from the United Nations FAO provided the only available global dietary estimates but with uncertain validity.

**Objectives:** We investigated how FAO estimates compared with nationally representative, individual-based dietary surveys from the Global Dietary Database (GDD) and developed calibration equations to improve the validity of FAO data to estimate dietary intakes.

**Design:** FAO estimates were matched to GDD data for 113 countries across the following 9 major dietary metrics for 30 y of data (1980–2009): fruit, vegetables, beans and legumes, nuts and seeds, whole grains, red and processed meats, fish and seafood, milk, and total energy. Both absolute and percentage differences in FAO and GDD mean estimates were evaluated. Linear regression was used to evaluate whether FAO estimates predicted GDD dietary intakes and whether this prediction varied according to age, sex, region, and time. Calibration equations were developed to adjust FAO estimates to approximate national dietary surveys validated by using randomly split data sets.

**Results:** For most food groups, FAO estimates substantially overestimated individual-based dietary intakes by 74.5% (vegetables) and 270% (whole grains) while underestimating beans and legumes (−50%) and nuts and seeds (−29%) (*P* < 0.05 for each). In multivariate regressions, these overestimations and underestimations for each dietary factor further varied by age, sex, region, and time (*P* < 0.001 for each). Split–data set calibration models, which accounted for country-level covariates and other sources of heterogeneity, effectively adjusted FAO estimates to approximate estimates from national survey data (*r* = 0.47–0.80) with small SEs of prediction (generally 1–5 g/d).

**Conclusions:** For all food groups and total energy, FAO estimates substantially exceeded or underestimated individual-based national surveys of individual intakes with significant variation depending on age, sex, region, and time. Calibration models effectively adjusted the comprehensive, widely accessible FAO data to facilitate a more-accurate estimation of individual-level dietary intakes nationally and by age and sex.

## INTRODUCTION

Accurate data on dietary intakes are essential to inform impacts on disease and public health priorities. However, few systematically assessed dietary intake data are available on a global scale. Until currently, estimates of national per capita food-supply availability (food balance sheets) from the United Nations FAO were widely used and cited by academics and government agencies to estimate national dietary intakes (e.g., to identify countries with lower-than-recommended dietary intakes, compare dietary intakes between countries and over time, construct estimates of undernourishment, and investigate the impact of specific dietary factors on health outcomes (1, 2).

Strengths of FAO food balance sheets include the inclusion of nearly all countries globally, use of reasonably comparable methods across countries, and open provision of accessible data sets. However, several limitations have constrained their usefulness for assessing dietary consumption. First, FAO estimates are derived by combining input variables for each food item, including total national production, total imports, total exports, total nonhuman use (e.g., livestock feed), and total waste from farms, distribution, and processing (3), each of which are subject to considerable error (3, 4). In addition, FAO estimates do not account for waste from cooking, spoilage, or plate waste; meals not eaten at home; home farming or production, which can be common in lower-income nations; and food reaching the household from nonretail markets. Analyses of a limited number of countries and food groups suggested that FAO estimates can dramatically overestimate national dietary consumption (5–7). For instance, in the United States, mean total energy intake was estimated to be 2081 kcal/d in the 2009–2010 NHANES (8) but 3688 kcal/d by using the corresponding FAO estimate (9). Finally, FAO data provide only national-level estimates per capita, which preclude the assessment of differences in intakes by key demographics such as age and sex.

To address these limitations, the Global Dietary Database (GDD) was assembled to systematically assess global dietary intakes on the basis of individual-level, nationally representative, nutritional survey data (10–12). The GDD contains quantitative estimates of the consumption of major foods and nutrients between 1980 and 2010 by country, including in 16 age- and sex-specific subgroups within each country. The primary aims of this work were to compare FAO estimates to GDD intake data and assess potential heterogeneity by dietary factor, age, sex, world region, and time. In addition, we determined whether calibration factors could be applied to FAO estimates to increase their correspondence with national dietary intake data, including observed differences by age and sex.

## METHODS

### Data sources

FAO annual food-supply data for 101 food items were obtained from standardized food balance sheets (http://faostat3.fao.org/faostat-gateway/go/to/download/FB/*/E) for all available nations and years between 1961 and 2009 (**Table 1**). Food-supply data represented the per capita quantity of food available for human consumption (3) calculated by the FAO by subtracting estimated use (quantity exported, fed to livestock, used for seed, processed for food use, and nonfood uses and losses during storage and transportation) from the estimated total supply (quantity imported and produced with adjustments for changes in stocks) (3, 4). Food balance sheet data were further standardized by converting processed commodities to a primary equivalent; e.g., quantities of bread are expressed in wheat equivalents comprising wheat flour and wheat-flour products (3). Data on most food items were available only as aggregates (e.g., poultry meat comprises chicken meat, turkey meat, and other poultry family meats) (3).

**TABLE 1:** Comparison of national-level dietary data available from the FAO and the GDD^1^

	FAO	GDD
Source of data	National per capita food supply available for consumption based on estimates of production, imports, exports, and changes in stock^2^	Individual-level data from national surveys
		Multiple dietary recalls and records (19%)
		Food-frequency questionnaires (22%)
		Single dietary recalls and records (30%)
		Household budget data from national surveys (24%)
Measure of data^3^	Mean per capita food or nutrient availability	Mean food or nutrient intake by age and sex groups
Countries covered, *n*	245	113
Population covered	100%	76% of global adult population
Surveys, *n*	NA	325
Individuals in surveys, *n*	NA	1,747,236
Years included, range	1961–2009	1980–2010
Representativeness	NA	National surveys (72%)
		Regional surveys (19%)
		Urban or rural; local or selected cohort (9%)
Foods, *n*	101 food items^4^	12 food categories (fruit, fruit juices, fruit and vegetables, vegetables, beans and legumes, nuts and seeds, whole grains, red meats, processed meats, fish and seafood, milk, and sugar-sweetened beverages)
Nutrients, *n*	3 (protein, fat, and energy)	10 (sodium, calcium, fiber, SFAs, *ω*-6 fatty acids, seafood *ω*-3 fatty acids, plant *ω*-3 fatty acids,* trans *fatty acids, cholesterol, and total energy)
Energy-adjusted estimates	No	Both unadjusted and energy-adjusted estimates
Measure of intake distribution	No	Yes (SD)
Sex-specific estimates	No	Yes
Age-specific estimates	No	Yes (8 age groups)

1 GDD, Global Dietary Database; NA, not applicable.

2 Calculated by the FAO as the residual from subtracting use (quantity exported, fed to livestock, used for seed, processed for food use and nonfood uses, and losses during storage and transportation) from the total supply (quantity imported and produced with adjustments for changes in stocks) divided by the population of a given nation.

3 GDD national means and means for age-sex subgroups were compared with FAO means for the analysis.

4 A list of FAO food items categorized to GDD food groups is available in Supplemental Table 1.

The GDD was assembled to quantitative estimates of dietary intakes of major foods and nutrients between 1980 and 2010 by world region, country, and 16 age- and sex-specific subgroups (Table 1). Detailed methods and results for data identification, acquisition, and selection were previously reported (10, 11, 13). In brief, systematic searches for nationally representative dietary surveys from around the world that assessed individual-level consumption were performed with mean intakes, SDs, and SEs assessed in ≤16 standardized age- and sex-specific groups for each survey as available. When national individual-level surveys were not identified for any country, large regional surveys were evaluated. Standardized methods were also used to extract and record evidence for selection bias, sample representativeness, response rate, sample size, and validity of dietary assessment methods. For the current analysis, we used the original (nonimputed), non–energy-adjusted data from each of the identified surveys.

### Data preparation

FAO food items were categorized and summed to correspond to each of 12 GDD food categories (**Supplemental Table 1**). For example, FAO estimates of groundnuts (shelled equivalent), sunflower seed, sesame seed, and tree nuts were summed to correspond to the GDD category nuts and seeds. Similarly, for FAO estimates corresponding to more than one GDD food category, GDD categories were combined (e.g., GDD categories fruit and fruit juices were summed, and GDD categories red meat and processed meat were summed. GDD national means were calculated on the basis of population-weighted means of separate age- and sex-specific estimates within each country. Differences in country names between FAO and GDD databases were manually remedied to ensure correspondence. FAO and GDD databases were merged and matched by country, food group, and year. For GDD means from multiyear surveys, an FAO average for corresponding years was assigned. At the national level, the final data set included 1145 matched pairs by food, nation, and year of FAO-GDD estimates and by age and sex in the GDD for a total of 7660 matched pairs.

### Statistical analysis

Characteristics of FAO and GDD data sets were first qualitatively evaluated. National means between FAO and GDD estimates were compared with the statistical significance of differences evaluated by using Wilcoxon’s signed-rank test. For each food group, the independent relation between FAO and GDD estimates was examined by using multivariate linear regression with robust variance estimators


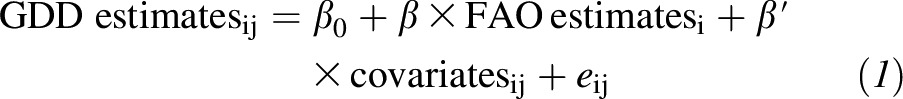


where GDD estimates_ij_ represent estimates of intake for country *i* and age- and sex-specific subgroup *j*; FAO estimates_i_ represent estimates of availability in country *i* matched to the GDD estimate_i_; and covariates_ij_ were covariates specific to country *i* and population subgroup *j*. Evaluated covariates included the time period of data collection (<2000, 2000–2004, and ≥2005), world region (North America, Western Europe, Latin America and Caribbean, Central Asia and Eastern and Central Europe, East and Southeast Asia, South Asia, North Africa and Middle East, Sub-Saharan Africa, and Australia and New Zealand), age (≤20 and 34, 35–49, ≥50 and 69, and ≥70 y), sex (men and women), dietary assessment method (repeated dietary recalls, food-frequency questionnaire, and single dietary recall), and survey representativeness (national, regional, and local/cohort). To assess potential heterogeneity in the association between GDD and FAO estimates, interaction terms were added to the multivariate model as cross-product terms of the mean FAO estimate (continuous) and each potential effect modifier (categorical as indicator variables). The statistical significance of each potential interaction was evaluated by using likelihood ratio testing and comparing the fully adjusted model that contained all interaction terms with a nested model in which each respective potential effect modifier was removed.

Calibration models were constructed on the basis of the variables included in the final multivariate model for each food group. The validity of each calibration model was performed by randomly assigning FAO-GDD data pairs to a derivation data set (60% of data) or validation data set (40% of data). In the derivation data set, an inverse-variance weighted multivariate linear regression was performed to provide derivation data set coefficients (β ± SE) for each term in the models. These coefficients were used to calculate predicted GDD means on the basis of FAO data, covariates, and interactions in the validation data set. The performance of the calibration was assessed by evaluating Spearman correlations, SEs of prediction, and mean squared errors between observed compared with predicted GDD means in the validation data set with the best fitting model by these metrics across food groups presented. Analyses were performed with STATA 12 software (StataCorp) with a 2-tailed α = 0.05.

## RESULTS

### Comparison of dietary data from FAO and GDD databases

FAO food balance sheets provided annual national per capita supply estimates of >100 foods, 2 macronutrients (protein and fat), and total energy for 245 nations between 1961 and 2009 (Table 1). In contrast, the GDD database provided national and age- and sex-specific dietary intake data for 13 food categories and 9 nutrients on the basis of individual-level data from 325 national or regional surveys that used 24-h diet recalls, 24-h records, or semiquantitative food-frequency questionnaires. FAO estimates provided no measures of data quality such as representativeness, bias, or validity; GDD data included assessments of selection bias, sample representativeness, response rate, sample size, and validity of dietary assessment methods. Overall, raw GDD data included fewer countries (*n* = 113) and years (1980–2010) than did the FAO database. The number of FAO-GDD matched data pairs by country and year varied across food groups from as many as 109 countries for fruit and vegetables to as few as 23 countries matched for whole grains (**Supplemental Table 2**). Unmatched country data were more common in Sub-Saharan Africa and the Caribbean and Latin America (**Supplemental Table 3**), which reflected the lack of individual-level national survey data in these regions.

### FAO estimates as predictors of GDD dietary intakes

**FIGURE 1:**
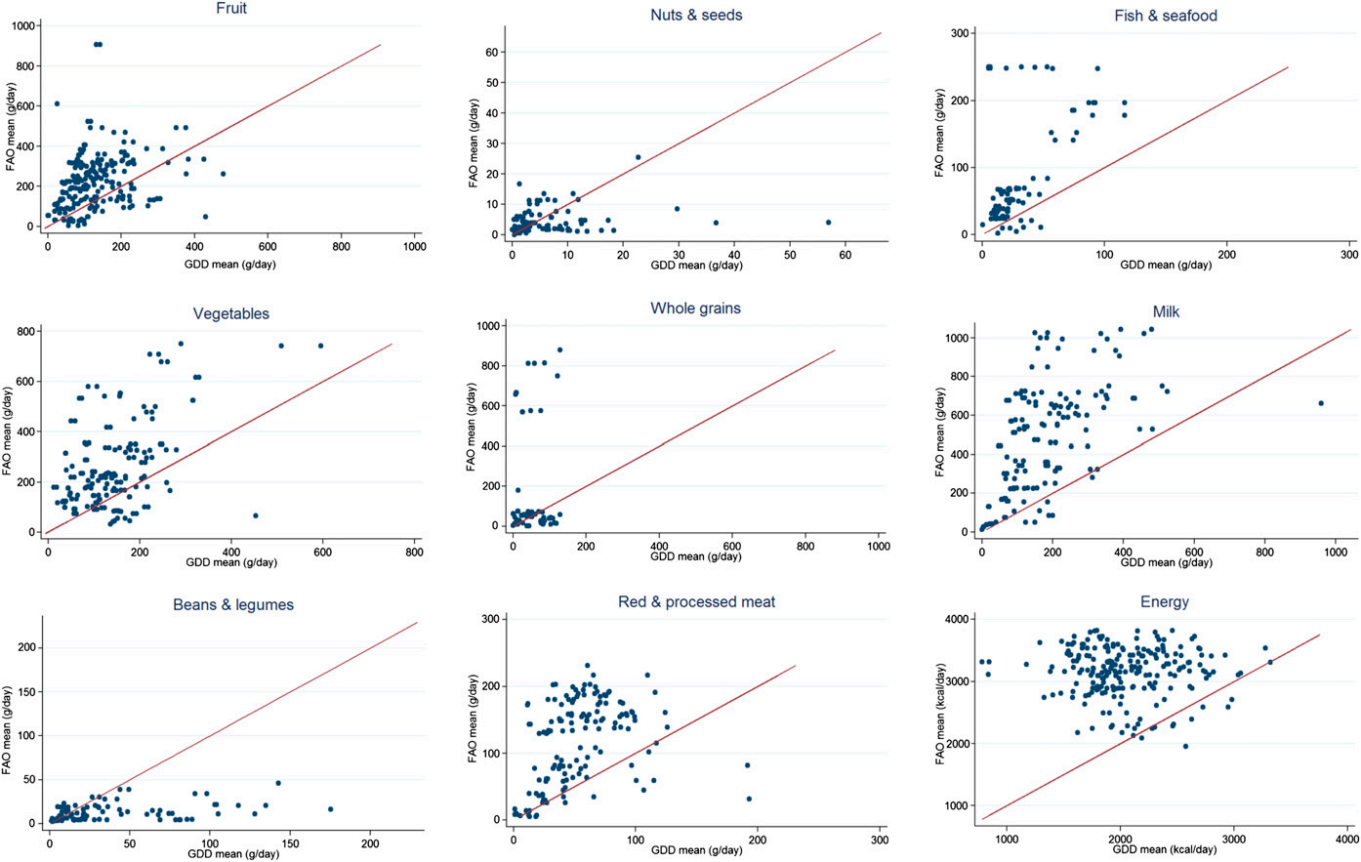
National GDD means compared with FAO means (95% CIs) (g/d), 1980–2009. FAO national food-supply estimates exceeded individual-based GDD national dietary intake estimates for most food groups, including fruit, vegetables, whole grains, red and processed meats, fish and seafood, milk, and total energy (*P* < 0.001 for each; most data points above 45 degree lines). FAO estimates significantly underestimated GDD intakes for beans and legumes (*P* < 0.001) and nuts and seeds (*P* < 0.05; most data points below 45 degree lines). Sample sizes of each food group are given in Table 2. GDD, Global Dietary Database.

FAO national food-supply estimates significantly exceeded individual-based GDD national dietary intake estimates for most food groups including fruit, vegetables, whole grains, red and processed meat, fish and seafood, milk, and total energy (*P* < 0.001 for each) (**Figure 1**, **Table 2**). The degree of overestimation ranged from 54% for total energy to 270% for whole grains. FAO estimates significantly underestimated GDD intakes for beans and legumes (*P* < 0.001) and nuts and seeds (*P* < 0.05).

**TABLE 2:** Comparisons of FAO and GDD means by food category (1980–2009)

		FAO-GDD pairs, *n*		Mean ± SD		Mean difference (FAO − GDD)		SE prediction^2^	
Food category	Countries,^1^ *n*	National	Age-sex^3^	FAO	GDD^4^	Values	%	Mean ± SD	Standardized mean
Fruit, g/d	109	189	1283	223 ± 133	125 ± 80	98	78.4	11.4 ± 3.9	0.09
Vegetables, g/d	109	135	838	260 ± 166	149 ± 85	111	74.5	20.7 ± 6.2	0.14
Beans and legumes, g/d	63	137	811	17 ± 14	34 ± 43	−17	−50.0	1.47 ± 1.0	0.04
Nuts and seeds, g/d	53	125	744	4.1 ± 3.4	5.8 ± 8.6	−1.7	−29.3	3.55 ± 3.5	0.61
Whole grains, g/d	25	33	448	185 ± 50	50 ± 37	135	270	117 ± 82	2.34
Red and processed meat, g/d	74	152	888	119 ± 66	54 ± 29	65	120	11.0 ± 7.8	0.20
Fish and seafood, g/d	46	107	557	78 ± 69	32 ± 26	46	144	6.43 ± 2.4	0.20
Milk, g/d	63	143	866	480 ± 285	176 ± 123	304	173	23.3 ± 8.8	0.13
Energy, kcal/d	59	124	1225	3131 ± 386	2031 ± 263	1100	54.1	50.0 ± 18.7	0.02

1 Listing of countries with FAO, GDD estimates, matched by year, for a given food category is provided in Supplementary Table 2. GDD, Global Dietary Database.

2 SE prediction is the SE of the predicted expected value for the observation’s covariate pattern. The mean ± SD for this statistic is provided as well as the standardized mean (mean SE of prediction ÷ GDD mean).

3 Age and sex-specific GDD mean estimates (up to 16 age-sex subgroups for a given nation and year) were matched to FAO national mean estimates.

4 Difference between FAO and GDD national means, *P* < 0.001 (Wilcoxon’s signed-rank test). FAO estimates substantially overestimated individual-based dietary intakes for all food groups except beans and legumes (*P* < 0.0001) and nuts and seeds (*P* < 0.05).

**FIGURE 2:**
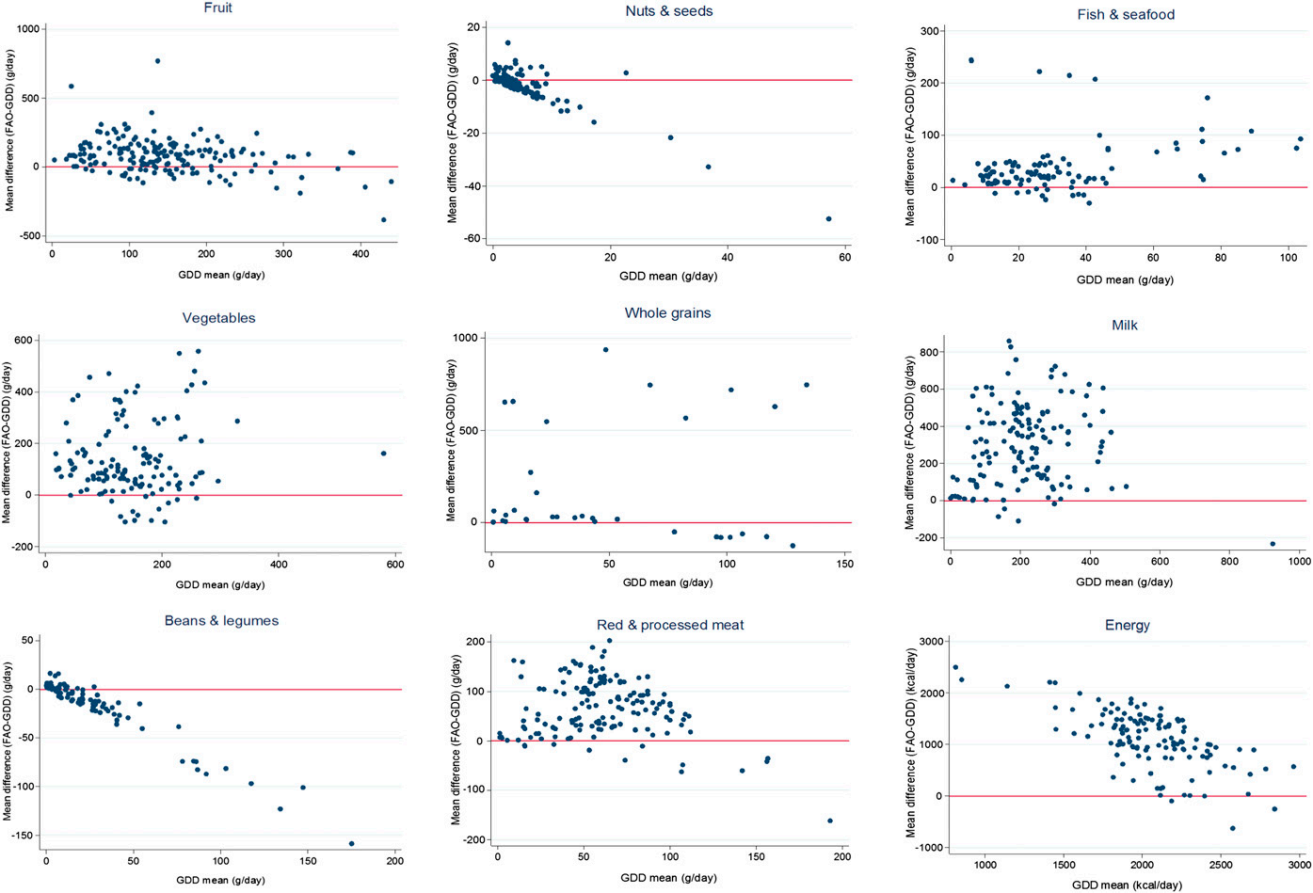
National GDD means compared with mean differences (FAO-GDD) (g/d), 1980–2009. For food groups underestimated by FAO data (beans and legumes; nuts and seeds), the discrepancy between FAO and GDD means was greater at higher absolute intakes, whereas for total energy, the overestimation was greater at lower absolute intakes. Sample sizes of each food group are given in Table 2. GDD, Global Dietary Database.

Variability was evident in the relation between FAO national estimates and GDD national data (Figure 1). For food groups underestimated by FAO data (beans and legumes; nuts and seeds), the discrepancy was greater at higher absolute intakes, whereas for total energy, the overestimation was greater at lower absolute intakes (*P* < 0.01; **Figure 2**). In comparison, for other food groups, the discrepancy between FAO estimates and GDD intakes was not strongly related to absolute intakes at the national level. In comparison, when evaluated by age and sex, trends toward a greater FAO overestimation at lower GDD values was observed for all food groups (**Supplemental Figure 1**).

In unadjusted linear regression models, FAO estimates were significant predictors of GDD estimates for all food groups (*P* < 0.001) except whole grains (*P* = 0.53) and total energy (*P* = 0.98) (**Table 3**). Modest proportions of the variance in GDD intakes were explained by FAO estimates for beans and legumes (*R*^2^ = 0.35), red and processed meats (*R*^2^ = 0.26), fish and seafood (*R*^2^ = 0.53), and milk (*R*^2^ = 0.32); the variation explained was weaker for the other food groups. Adjustment for age and sex had little influence on relations between FAO estimates and GDD data. However, additional adjustment for world region, period of data collection, GDD survey representativeness, and GDD dietary assessment method attenuated the relation for nuts and seeds and improved the relation for whole grains and total energy.

**TABLE 3:** Relation between FAO food-availability estimates as predictors of GDD mean dietary intakes in all age-sex groups^1^

Food category and model	Intercept ± SE	β (FAO) ± SE	*P*	*R*^2^
Fruit, g/d			
Unadjusted	85.8 ± 4.3	0.18 ± 0.02	<0.001	0.09
Age and sex	76.1 ± 5.6	0.18 ± 0.02	<0.001	0.09
Multivariate	64.9 ± 7.0	0.07 ± 0.01	<0.005	0.35
Vegetables, g/d				
Unadjusted	95.7 ± 5.1	0.12 ± 0.01	<0.001	0.12
Age and sex	87.0 ± 6.4	0.12 ± 0.01	<0.001	0.12
Multivariate	148.6 ± 11.1	0.10 ± 0.01	<0.001	0.33
Beans and legumes, g/d				
Unadjusted	4.7 ± 2.5	2.42 ± 0.24	<0.001	0.35
Age and sex	6.1 ± 3.7	2.43 ± 0.24	<0.001	0.35
Multivariate	32.7 ± 4.7	2.39 ± 0.40	<0.001	0.58
Nuts and seeds				
Unadjusted	4.32 ± 0.3	0.38 ± 0.07	<0.001	0.02
Age and sex	4.21 ± 0.7	0.39 ± 0.07	<0.001	0.35
Multivariate	8.12 ± 1.6	0.17 ± 0.14	0.23	0.37
Whole grains				
Unadjusted	48.8 ± 2.1	0.01 ± 0.01	0.53	0.01
Age and sex	48.5 ± 3.5	0.01 ± 0.01	0.53	0.01
Multivariate	0.92 ± 6.4	0.01 ± 0.01	0.027	0.62
Red and processed meats, g/d				
Unadjusted	27.1 ± 1.8	0.24 ± 0.01	<0.001	0.26
Age and sex	25.5 ± 2.2	0.23 ± 0.01	<0.001	0.27
Multivariate	35.7 ± 4.2	0.22 ± 0.03	<0.001	0.50
Fish and seafood, g/d				
Unadjusted	10.3 ± 1.1	0.27 ± 0.02	<0.001	0.53
Age and sex	5.9 ± 1.4	0.27 ± 0.02	<0.001	0.55
Multivariate	43.0 ± 2.2	0.14 ± 0.02	<0.001	0.84
Milk, g/d				
Unadjusted	58.5 ± 4.4	0.25 ± 0.01	<0.001	0.32
Age and sex	75.0 ± 7.6	0.24 ± 0.01	<0.001	0.33
Multivariate	−17.9 ± 11.4	0.24 ± 0.03	<0.001	0.51
Energy (kcal/d)				
Unadjusted	2029.0 ± 57.8	<0.01 ± 0.02	0.98	<0.01
Age and sex	1994.7 ± 60.1	0.01 ± 0.02	0.78	0.01
Multivariate	1676.3 ± 140.2	0.12 ± 0.05	0.016	0.22

1 On the basis of linear regression models with FAO estimates as the dependent variable and GDD estimates as the independent variable. The age and sex model was categorized as follows: age, ≤20 and 34, 35–49, ≥50 and 69, and ≥70 y; sex, M and F. The multivariate model was adjusted for the following covariates: age (≤20 and 34, 35–49, ≥50 and 69, and ≥70 y), sex (M and F), assessment method [less than one dietary recall, food-frequency questionnaire, and one dietary recall), region (North America, Western Europe, Latin America and Caribbean, Central Asia and Eastern and Central Europe, East and Southeast Asia, South Asia, North Africa and Middle East, Sub-Saharan Africa, and Australia and New Zealand), starting year of data collection (<2000, 2000–2004, and ≥2005), and representativeness (national, regional, or local/cohort) as covariates. β(FAO) represents the change in the GDD mean for a 1-g/d increase in the FAO mean. Robust SEs for β (FAO) and the intercept are presented. *R*^2^ represents the coefficient of determination for the overall model. GDD, Global Dietary Database.

When various characteristics were separately evaluated, a significant independent heterogeneity was identified in the relation between FAO estimates and GDD intakes for many food groups according to age, sex, world region, time period of data collection, and survey assessment method and representativeness (**Table 4**). For example, for every food group evaluated, the relation between FAO estimates and GDD intakes varied by world region (*P* < 0.01 for each). In contrast, for total energy, the relation only significantly varied by world region and the time period of data collection.

**TABLE 4:** Evidence for variation in the multivariate relation of FAO food-availability estimates as predictors of GDD mean dietary intakes^1^

	*P*-interaction					
Food category	Sex	Age	Region	Year	Assessment	Representativeness
Fruit, g/d	0.85	<0.01	<0.01	<0.01	<0.01	<0.01
Vegetables, g/d	0.04	0.82	<0.01	<0.01	<0.01	<0.01
Beans and legumes, g/d	<0.01	<0.01	<0.01	0.14	<0.01	0.20
Nuts and seeds, g/d	0.16	<0.01	<0.01	0.98	<0.01	<0.01
Whole grains, g/d	0.64	0.80	<0.01	<0.01	<0.01	<0.01
Red and processed meats, g/d	<0.01	0.30	<0.01	0.23	<0.01	0.13
Fish and seafood, g/d	<0.01	<0.01	<0.01	<0.01	<0.01	<0.01
Milk, g/d	<0.01	<0.01	<0.01	0.53	<0.01	<0.01
Energy, kcal/d	<0.01	0.18	<0.01	0.04	0.34	0.25

1 Multiplicative interaction terms for each potential effect modifier (sex, age, region, assessment method, and representativeness) were constructed and added to the fully adjusted model, which included age (≤20 and 34, 35–49, ≥50 and 69, and ≥70 y), sex (only M and only F), assessment method (less than one dietary recall, food-frequency questionnaire, one dietary recall, or household-availability data), region (North America, Western Europe, Latin America and Caribbean, Central Asia and Eastern and Central Europe, East and Southeast Asia, South Asia, North Africa and Middle East, Sub-Saharan Africa, and Australia and New Zealand), starting year of data collection (<2000, 2000–2004, and ≥2005), and representativeness (national, regional, or local/cohort) as covariates. The fully adjusted model that contained all interaction terms was compared by using the likelihood ratio test to a nested model in which each respective interaction term was removed. GDD, Global Dietary Database.

### Calibration

**FIGURE 3:**
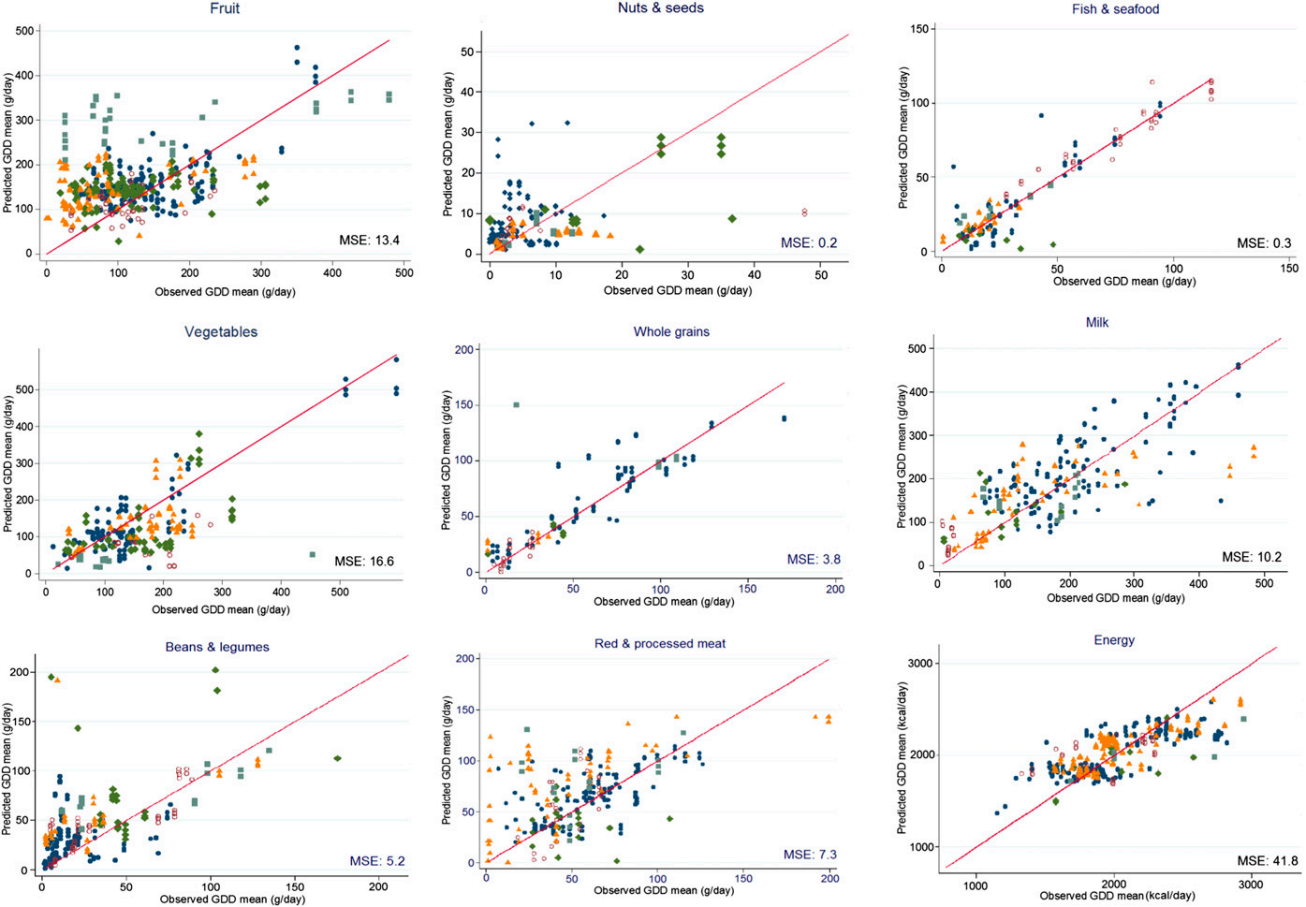
Observed compared with predicted GDD means from split–data set calibration modeling. For each food group, the β (±SE) for each term included in the models for the age-sex data set is given in Supplemental Table 4. The MSE represents the average of squares of the difference between observed and predicted GDD means in the validation data set. Calibration models effectively adjusted FAO estimates to approximate estimates from national survey data with generally small MSEs. Filled navy blue circles denote North America, Western Europe, Australia, and New Zealand. Open red circles denote Southeast Asia and Asia Pacific. Orange triangles denote Central Asia and Eastern and Central Europe. Light blue squares denote Latin America, the Caribbean, and Oceania. Green diamonds denote Sub-Saharan Africa, North Africa, and the Middle East. GDD, Global Dietary Database; MSE, mean squared error.

In a randomly selected derivation data set (60% of data), multivariate-adjusted relations between FAO estimates and GDD data were evaluated with final models and coefficients from the derivation data set presented in **Supplemental Table 4**. When these coefficients were applied to the validation data set, good agreement between observed compared with predicted GDD means was observed (**Figure 3**, **Table 5**). The intercorrelation was lower for whole grains (0.47) and red and processed meats (0.48) but still with reasonably small SEs of prediction (9.9 ± 2.6 and 11.2 ± 3.0, respectively). Correlations between observed compared with predicted GDD intakes were higher (0.60–0.80) for other food groups with reasonably small SEs of prediction and mean squared errors.

**TABLE 5:** Relation of observed compared with predicted GDD data from calibration modeling^1^

	Validation set^2^			SE prediction^4^		
Food category	Observed mean	Predicted mean^6^	Spearman’s ρ^3^	Mean ± SD	Standardized mean	MSE^5^
Fruit, g/d	124 ± 83	123 ± 57	0.60	2.55 ± 0.91	0.02	13.4
Vegetables, g/d	148 ± 96	146 ± 76	0.63	3.24 ± 1.23	0.02	16.6
Beans and legumes, g/d	36 ± 50	33 ± 48	0.69	3.16 ± 1.92	0.09	5.2
Nuts and seeds, g/d	5.9 ± 9.3	4.9 ± 31	0.62	2.20 ± 1.21	0.37	0.2
Whole grains, g/d	47 ± 39	32 ± 98	0.47	9.87 ± 2.56	0.21	3.8
Red and processed meat, g/d	53 ± 34	71 ± 33	0.48	11.21 ± 3.04	0.21	7.3
Fish and seafood, g/d	31 ± 27	31 ± 27	0.80	0.94 ± 0.35	0.02	0.3
Milk, g/d	171 ± 135	198 ± 114	0.78	5.63 ± 2.42	0.03	10.2
Energy, kcal/d	1969 ± 405	1961 ± 275	0.69	12.6 ± 4.30	0.01	41.8

1 Calibration models were built by randomly assigning data pairs for a given food group to a derivation data set (60% of data) or validation data set (40% of data). In the derivation set, a multivariate linear regression of FAO estimates as predictors of GDD mean dietary intakes was specified by using the following adjusted model including age (≤20 and 34, 35–49, ≥50 and 69, and ≥70 y), sex (only M and only F), assessment method (less than one dietary recall, food-frequency questionnaire, one dietary recall, or household-availability data), region (Southeast and Asia Pacific, North America, Australia and New Zealand, Western Europe, Sub-Saharan Africa, North Africa and Middle East, Central Asia and Eastern and Central Europe, Latin America, Caribbean, and Oceania), starting year of data collection (<2000, 2000–2004, and ≥2005), and representativeness (national, regional, or local/cohort) as covariates in addition to multiplicative interaction terms for age, sex, assessment method, year of data collection, and representativeness. For each food group, the β ± SE for each term included in models for the full age-sex data set is given in Supplementary Table 4. Coefficients obtained from derivation data set models were used to calculate the predicted GDD mean in the validation set. GDD, Global Dietary Database; MSE, mean squared error.

2 Values are means ± SDs.

3 Spearman’s rank-order correlation of the association of the validation set observed GDD mean and validation set GDD mean for a given food group is provided. All correlations were significant at *P* < 0.001.

4 SE prediction is the SE of the predicted expected value for observations. The mean ± SD for this statistic is provided as well as the standardized mean (mean SE of prediction ÷ GDD mean)

5 MSE represents the average of squares of the difference between observed GDD means and predicted GDD means in the validation data set.

6 Validation set GDD means were calculated from models developed in derivation data sets.

## DISCUSSION

In this systematic comparison of 30 y of FAO food-supply estimates with all available nationally representative dietary survey data collected in the GDD over the same time period, we showed that FAO estimates substantially exceeded GDD national survey intakes by between 75% and 270% for major food groups. This overestimation was not consistent but varied by world region, sex, age, and time period. With the use of country-level covariates, we showed that it was possible to calibrate FAO data estimates to achieve a close fit between predicted dietary intake estimates and actual estimates provided by nationally representative dietary surveys.

Our findings of a substantial FAO overestimation of individual-level dietary intakes for most food groups were consistent with methods used to generate FAO data, which were developed to capture food availability rather than actual intake, do not account for all sources of waste (4), and can underestimate the resident population used to calculate per capita values (14). Our results corroborate and greatly expand on previous reports, which compared FAO food-supply data to individual dietary data for only a few countries (14, 15) or selected dietary components such as fruit and vegetables (16). Overestimation was greatest for whole grains even though we attempted to minimize the lack of available conversion and processing factors within FAO food balance sheets by removing the major categories of rice, wheat, and other low-fiber grains from the FAO grouping. In contrast to most foods, the FAO underestimated values for nuts and seeds and beans and legumes. This underestimation could have been due to home or local production, meals not eaten at home, or other sources not captured by the FAO. Our findings highlight the need to investigate the reasons behind these overestimations and underestimations of dietary intakes, particularly in regions and age and sex groups with the largest discrepancies.

Although the FAO suggests several appropriate uses for its food-supply estimates, including the observation of a country’s food supply and trends over time, and projections of food supply (4, 17–19), our findings showed that the use of FAO estimates to assess dietary quality or to examine diet-disease burden relations (20, 21) is highly problematic. Conversely, FAO food balance sheets include all major countries in the world, are available annually, and are easily accessible, each of which is a key strength for global analyses. In light of these issues, our calibration equations provide an accessible, effective way to adjust FAO estimates so that they may be used to approximate survey intakes nationally as well as by age and sex.

It is important to highlight that GDD data are also imperfect; for instance, less data are available in certain world regions, data are only available for broad food categories, and assessment methods vary among sources of dietary data. Despite these limitations, data from representative, individual-level dietary surveys remain the best practical standard for the assessment of dietary consumption, dietary quality (22), or diet-disease burden relations (11, 13, 23) globally and across nations.

Our analysis had several strengths. To our knowledge, our methods represent the first effort to quantify the differences in FAO food-supply data and dietary intake data from national surveys on a global scale, with 109 countries represented. Our novel calibration equations can be applied to FAO food-supply estimates to approximate dietary intake data. This method allowed us to take advantage of GDD data representative of dietary intakes both nationally and by age and sex groupings, but not available for all countries and years, to calibrate FAO estimates, which are available for all major countries and years and widely accessible, but with limitations of inaccuracies in estimation, to derive models to more-accurately estimate individual-level dietary intakes.

Finally, common gaps and limitations exist in the nutrient data available from both the FAO and GDD. FAO food balance sheets provide per capita data for only 2 macronutrients (protein and fat) and total energy, whereas the GDD is currently comprised of only 12 major food groups, 9 nutrients, and total energy, with a focus on factors linked to chronic diseases such as cardiovascular disease and cancers. Neither the FAO nor GDD provides estimates of micronutrients relevant for deficiency conditions, undernourishment assessment, or infant-child outcomes (stunting, anemia, and mortality), such as vitamin A, zinc, iodine, and iron for key population subgroups of interest, such as children, adolescents, and pregnant and nursing mothers. Such data are of key importance in devising evidence-based priorities for policies and prevention globally and enhancing or supplementing undernourishment assessment indexes to include diet-disease considerations in addition to total energy (24). Over the next 3 y, we will be updating the GDD to include nutrients relevant for deficiency conditions and maternal-child health, including vitamin A, iron, B vitamins, zinc, iodine, selenium, and other micronutrients, and compiling available data within nations by urban and rural locations and income status, up to the year 2015 (25). Although limitations in the updated GDD will still reflect data gaps in national or subnational dietary survey data available worldwide, the updated database will provide the most-comprehensive assessment of intakes of major food groups and nutrients relevant to deficiency diseases for informing priorities for policies and prevention strategies in the poorest and most-vulnerable populations around the world.

In conclusion, we showed that FAO food-supply estimates substantially overestimated or underestimated dietary intakes from individual-based national surveys worldwide with significant heterogeneity in this overestimation or underestimation according to age, sex, region, and time. Calibration models constructed in this analysis by using FAO food-supply data and country-level covariates effectively adjusted FAO estimates and improve their validity for estimating dietary intakes at the national level and by age and sex. Given the other advantages of the FAO database, these findings will facilitate future use of FAO estimates by scientists and policy makers to better approximate dietary intakes. This calibration will provide the best estimates for the accurate assessment of dietary consumption, dietary quality, diet-disease burden relations, and relevant policy.

## Supplementary Material

Supplemental data
