# Supplementary material for: Assessing global dietary habits: a comparison of national estimates from the FAO and the Global Dietary Database1
Source: Am J Clin Nutr. 2015 Mar 18;101(5):1038–46. doi: 10.3945/ajcn.114.087403 (PMC4409685; doi:10.3945/ajcn.114.087403)
Supplement: Supplemental data [file supp_101_5_1038__index.html]

Supplemental data 

# Assessing global dietary habits: a comparison of national estimates from the FAO and the Global Dietary Database

## Supplemental data

**Files in this Data Supplement:**

- Supplemental data - Tables 1-4 and Figure 1
