# Supplementary material for: Assessing global dietary habits: a comparison of national estimates from the FAO and the Global Dietary Database1
Source: Am J Clin Nutr. 2015 Mar 18;101(5):1038–46. doi: 10.3945/ajcn.114.087403 (PMC4409685; doi:10.3945/ajcn.114.087403)
Supplement: Supplemental data [file 114.087403_ajcn087403SupplementaryData1.docx]

**Supplementary Table 1**. Matching of FAO items to GDD food categories

| GDD group(s) | FAO item | FAO definition^1^ |
| --- | --- | --- |
| **Fruits** (& Fruit Juices)  Includes all listed FAO fruit items  Includes GDD Fruit Juice data | Coconuts - Incl Copra  Plantains  Bananas  Oranges, Mandarines  Lemons, Limes  Grapefruit  Citrus, Other  Apples  Grapes  Pineapples  Dates  Fruits, Other | Default composition: 249 Coconuts, 250 Coconuts Desiccated, 251 Copra  Default composition: 489 Plantains  Default composition: 486 Bananas  Default composition: 490 Oranges, 491 Orange juice, single strength, 492 Orange juice, concentrated, 495 Tangerines, mandarins, clem., 496 Tangerine Juice  Default composition: 497 Lemons and limes, 498 Lemon juice, single strength, 499 Lemon juice, concentrated  Default composition: 507 Grapefruit (inc. pomelos), 509 Juice of Grapefruit, 510 Grapefruit juice, concentrated  Default composition: 512 Citrus fruit, nes, 513 Citrus juice, single strength, 514 Citrus juice, concentrated  Default composition: 515 Apples, 518 Apple juice, single strength, 519 Apple juice, concentrated  Default composition: 560 Grapes, 561 Raisins, 562 Grape Juice, 563 Must of Grapes  Default composition: 574 Pineapples, 575 Pineapples Cand, 576 Juice of Pineapples, 580 Pineapple Juice Conc  Default composition: 577 Dates  Default composition: 521 Pears, 523 Quinces, 526 Apricots, 527 Dry Apricots, 530 Sour cherries, 531 Cherries, 534 Peaches and nectarines, 536 Plums and sloes, 537 Plums Dried (Prunes), 538 Plum juice, single strength, 539 Plum juice, concentrated, 541 Stone fruit, nes, 542 Pome fruit, nes, 544 Strawberries, 547 Raspberries, 549 Gooseberries, 550 Currants, 552 Blueberries, 554 Cranberries, 558 Berries Nes, 567 Watermelons, 568 Other melons (inc.cantaloupes), 569 Figs, 570 Figs Dried, 571 Mangoes, mangosteens, guavas, 572 Avocados, 583 Mango Juice, 587 Persimmons, 591 Cashewapple, 592 Kiwi fruit, 600 Papayas, 603 Fruit, tropical fresh nes, 604 Fruit Tropical Dried Nes, 619 Fruit Fresh Nes, 620 Fruit Dried Nes, 622 Fruit Juice Nes, 623 Fruit Prp Nes, 624 Flour of Fruits, 625 Fruit,Nut,Peel, Sugar Prs, 626 Homogen. Cooked Fruit Prp |
| **Vegetables** (excludes starchy vegetables)  Includes onions, tomatoes, vegetables (other), aquatic vegetables | Onions  Tomatoes  Vegetables, Other  Aquatic  vegetables | Default composition: 403 Onions, dry  Default composition: 388 Tomatoes, 389 Tomatojuice Concentrated, 390 Juice of Tomatoes, 391 Paste of Tomatoes, 392 Tomato Peeled  Default composition: 358 Cabbages and other brassicas, 366 Artichokes, 367 Asparagus, 372 Lettuce and chicory, 373 Spinach, 378 Cassava leaves, 393 Cauliflowers and broccoli, 394 Pumpkins, squash and gourds, 397 Cucumbers and gherkins, 399 Eggplants (aubergines), 401 Chillies and peppers, green, 402 Onions (inc. shallots), green, 406 Garlic, 407 Leeks, other alliaceous veg, 414 Beans, green, 417 Peas, green, 420 Leguminous vegetables, nes, 423 String beans, 426 Carrots and turnips, 430 Okra, 446 Maize, green, 447 Sweet Corn Frozen, 448 Sweet Corn Prep or Preserved, 449 Mushrooms and truffles, 450 Dried Mushrooms, 451 Canned Mushrooms, 459 Chicory roots, 461 Carobs, 463 Vegetables fresh nes, 464 Vegetables, dried nes, 465 Vegetables, canned nes, 466 Juice of Vegetables Nes, 469 Vegetables Dehydrated, 471 Vegetables in Vinegar, 472 Vegetables Preserved Nes, 473 Vegetable Frozen, 474 Veg.in Tem. Preservatives, 475 Veg.Prep. Or Pres.Frozen, 476 Homogen.Veget.Prep, 567 Watermelons, 568 Other melons (inc.cantaloupes), 658 Coffee Subst. Cont.Coffee  Default composition: 1594 Aquatic plants, fresh, 1595 Aquatic plants, dried, 1596 Aquatic plants, other preparations |
|  |  |  |
| **Beans & legumes**  Includes beans, peas, pulses, pulses (other), soybeans | Beans  Peas  Pulses  Pulses, Other  Soybeans | Default composition: 176 Beans, dry  Default composition: 187 Peas, dry  Default composition: 181 Broad beans, horse beans, dry, 191 Chick peas, 195 Cow peas, dry, 197 Pigeon peas, 201 Lentils, 203 Bambara beans, 205 Vetches, 210 Lupins, 211 Pulses, nes, 212 Flour of Pulses; nutrient data only: 213 Bran of Pulses  -  Default composition: 236 Soybeans, 239 Soya Sauce, 240 Soya Paste, 241 Soya Curd |
| **Nuts & seeds**  Includes all listed FAO nuts & seeds | Groundnuts (Shelled Eq)  Sunflower seed  Sesame seed  Treenuts | -  Default composition: 267 Sunflower seed  Default composition: 289 Sesame seed  Default composition: 216 Brazil nuts, with shell, 217 Cashew nuts, with shell, 220 Chestnuts, 221 Almonds, with shell, 222 Walnuts, with shell, 223 Pistachios, 224 Kolanuts, 225 Hazelnuts, with shell, 226 Arecanuts, 229 Brazil Nuts Shelled, 230 Cashew Nuts Shelled, 231 Almonds Shelled, 232 Walnuts Shelled, 233 Hazelnuts Shelled, 234 Nuts, nes, 235 Prepared Nuts (Exc.Groundnuts) |
| **Whole grains** (includes high fiber grains)  Includes rye, barley, oats, maize, cereals (other), sorghum, millet | Rye  Barley  Oats  Maize (corn)  Cereals, other  Sorghum  Millet | Default composition: 71 Rye, 72 Flour of Rye; nutrient data only: 73 Bran of Rye  Default composition: 44 Barley, 45 Pot Barley, 46 Barley Pearled, 49 Malt, 50 Malt Extract; nutrient data only: 47 Bran of Barley, 48 Barley Flour and Grits  Default composition: 75 Oats, 76 Oats Rolled; nutrient data only: 77 Bran of Oats  Default composition: 56 Maize, 58 Flour of Maize, 64 Starch of Maize, 846 Gluten Feed and Meal; nutrient data only: 57 Germ of Maize, 59 Bran of Maize, 63 Maize gluten  Default composition: 68 Popcorn, 89 Buckwheat, 90 Flour of Buckwheat, 92 Quinoa, 94 Fonio, 95 Flour of Fonio, 97 Triticale, 98 Flour of Triticale, 101 Canary seed, 103 Mixed grain, 104 Flour of Mixed Grain, 108 Cereals, nes, 111 Flour of Cereals, 113 Cereal Preparations, Nes; nutrient data only: 91 Bran Buckwheat, 96 Bran of Fonio, 99 Bran of Triticale, 105 Bran of Mixed Grains, 112 Bran of Cereals  Default composition: 83 Sorghum, 84 Flour of Sorghum; nutrient data only: 85 Bran of Sorghum  Default composition: 79 Millet, 80 Flour of Millet; nutrient  data only: 81 Bran of Millet |
| **Red & processed meat**  Includes all listed FAO meats. Excludes offals | Pigmeat  Mutton & Goat Meat  Bovine Meat  Meat, Other | Default composition: 1035 Pig meat, 1038 Pork, 1039 Bacon and Ham, 1041 Sausages of Pig Meat, 1042 Prep of Pig  Default composition: 977 Sheep meat, 1017 Goat meat  Default composition: 867 Cattle meat, 870 Meat-CattleBoneless(Beef and Veal), 872 Meat of Beef,Drd, Sltd,Smkd, 873 Meat Extracts, 874 Sausage Beef and Veal, 875 Preparations of Beef Meat, 876 Beef canned, 877 Homogen.Meat Prp., 947 Buffalo meat  Default composition: 1089 Bird meat, nes, 1097 Horse meat, 1108 Meat of Asses, 1111 Meat of Mules, 1127 Camel meat, 1141 Rabbit meat, 1151 Meat of Other Rod, 1158 Meat Oth Camelids, 1163 Game meat, 1164 Meat Dried Nes, 1166 Meat nes, 1172 Prepared Meat Nes, 1176 Snails, Not Sea |
|  |  |  |
| **Fish & seafood**  Includes all listed FAO fish & seafood  **Milk**  **Energy** | Freshwater Fish  Demersal Fish  Pelagic Fish  Crustaceans  Molluscs, Other  Aquatic Animals, Others  Marine Fish, Other  Cephalopods  Fish, Seafood  Aquatic Products, Other  Milk, excluding butter  Grand total (kcal/day) | Default composition: 1501 Frwtr Diad F, 1502 Frwtr Fz Whl, 1503 Frwtr Fillet, 1504 Frwtr Fz Flt, 1505 Frwtr Cured, 1506 Frwtr Canned, 1507 Frwtr Pr nes, 1508 Frwtr Meals  Default composition: 1514 Dmrsl Fresh, 1515 Dmrsl Fz Whl, 1516 Dmrsl Fillet, 1517 Dmrsl Fz Flt, 1518 Dmrsl Cured, 1519 Dmrsl Canned, 1520 Dmrsl Pr nes, 1521 Dmrsl Meals  Default composition: 1527 Pelagic Frsh, 1528 Pelgc Fz Whl, 1529 Pelgc Fillet, 1530 Pelgc Fz Flt, 1531 Pelgc Cured, 1532 Pelgc Canned, 1533 Pelgc Pr nes, 1534 Pelgc Meals  Default composition: 1553 Crstaceans F, 1554 Crstc Frozen, 1555 Crstc Cured, 1556 Crstc Canned, 1557 Crstc Pr nes, 1558 Crstc Meals  Default composition: 1562 Mlluscs Frsh, 1563 Molsc Frozen, 1564 Molsc Cured, 1565 Molsc Canned, 1566 Molsc Meals  Default composition: 1587 Aqutc Anim F, 1588 Aq A Cured, 1589 Aquatic Animals Meals, 1590 Aq A Prep Ns  Default composition: 1540 Marine nes F, 1541 Marin Fz Whl, 1542 Marin Fillet, 1543 Marin Fz Flt, 1544 Marin Cured, 1545 Marin Canned, 1546 Marin Pr nes, 1547 Marin Meals  Default composition: 1570 Cephlp Fresh, 1571 Cphlp Frozen, 1572 Cphlp Cured, 1573 Cphlp Canned, 1574 Cphlp Pr nes, 1575 Cphlp Meals    -  - |

^1^Descriptions were obtained from the FAO directory for definitions and classifications of commodities, available online: http://www.fao.org/waicent/faoinfo/economic/faodef/faodefe.htm#COMG

**Supplementary Table 2.** List of countries with both FAO and GDD estimates available by food category

| **Countries** | **Fruits** | **Vegetables** | **Beans & legumes** | **Nuts & seeds** | **Whole grains** | **Red & processed meat** | **Fish & seafood** | **Milk** | **Energy (kcal)** |
| --- | --- | --- | --- | --- | --- | --- | --- | --- | --- |
| Algeria  Argentina  Armenia  Australia  Austria  Bangladesh  Barbados  Belgium  Benin  Bosnia & Herzegovina  Botswana  Brazil  Bulgaria  Burkina Faso  Cambodia  Cameroon  Canada  Cape Verde  Chad  China  Colombia  Congo  Croatia  Cyprus  Czechoslovakia  Denmark  Egypt  Eritrea  Estonia  Ethiopia  Finland  France  Gabon  Georgia  Germany  Greece  Hungary  Iceland  India  Indonesia  Iran  Ireland  Israel  Italy  Jamaica  Japan  Jordan  Kazakhstan  Kuwait  Kyrgyzstan  Latvia  Lao PDR  Lebanon  Libya  Lithuania  Luxembourg  Madagascar  Malawi  Malaysia  Mali  Malta  Mauritania  Mexico  Moldova  Mongolia  Montenegro  Mozambique  Nepal  Netherlands  New Zealand  Niger  Norway  Pakistan  Philippines  Poland  Portugal  Republic of Korea  Romania  Russian Federation  Samoa  Serbia  Sierra Leone  Slovakia  Slovenia  Solomon Islands  South Africa  Spain  Sri Lanka  Swaziland  Sweden  Switzerland  Thailand  Tonga  Trinidad & Tobago  Turkey  Ukraine  United Kingdom  United States of America  Uruguay  Vanuatu  Zambia | X  X X X X X X X X  X X X X X  X X X X X X X  X X X X  X X X X X X X X X X X X X X  X X X X X X X X X  X X  X X X  X  X  X X X X X X  X  X  X X X X X X X X X X X X  X  X X X X  X X  X X X  X X X X X X  X  X X  X  X  X X X | X  X  X  X  X  X  X  X  X  X  X  X  X  X  X  X  X  X  X  X  X  X  X  X  X  X  X  X  X  X  X  X  X  X  X  X  X  X  X  X  X  X  X  X  X  X  X  X  X  X  X  X  X  X  X  X  X  X  X  X  X  X  X  X  X  X  X  X  X  X  X  X  X  X  X  X  X  X  X  X  X  X  X  X  X  X  X  X  X  X  X  X  X  X  X  X  X  X  X | X  X  X  X  X  X  X  X  X  X  X  X  X  X  X  X  X  X  X  X  X  X  X  X  X  X  X  X  X  X  X  X  X  X  X  X  X  X  X  X  X  X  X  X  X  X  X  X  X  X  X  X  X  X  X  X  X  X  X  X | X  X  X  X  X  X  X  X  X  X  X  X  X  X  X  X  X  X  X  X  X  X  X  X  X  X  X  X  X  X  X  X  X  X  X  X  X  X  X  X  X  X  X  X  X  X  X  X  X  X | X  X  X  X  X  X  X  X  X  X  X  X  X  X  X  X  X  X  X  X  X  X | X  X  X  X  X  X  X  X  X  X  X  X  X  X  X  X  X  X  X  X  X  X  X  X  X  X  X  X  X  X  X  X  X  X  X  X  X  X  X  X  X  X  X  X  X  X  X  X  X  X  X  X  X  X  X  X  X  X  X  X  X  X  X  X  X  X  X  X  X  X  X | X  X  X  X  X  X  X  X  X  X  X  X  X  X  X  X  X  X  X  X  X  X  X  X  X  X  X  X  X  X  X  X  X  X  X  X  X  X  X  X  X  X  X  X  X  X | X  X  X  X  X  X  X  X  X  X  X  X  X  X  X  X  X  X  X  X  X  X  X  X  X  X  X  X  X  X  X  X  X  X  X  X  X  X  X  X  X  X  X  X  X  X  X  X  X  X  X  X  X X  X  X  X  X  X  X  X  X  X | X  X  X  X  X  X  X  X  X  X  X  X  X  X  X  X  X  X  X  X  X  X  X  X  X  X  X  X  X  X  X  X  X  X  X  X  X  X  X  X  X  X  X  X  X  X  X  X  X  X  X  X  X  X  X  X  X  X  X |

**Supplementary Table 3.** Country-level data only available in the FAO database (n=67) or the GDD database (n=15) (unmatched country data)^1^

| FAO only | | GDD only |
| --- | --- | --- |
| Albania  Angola  Antigua & Barbuda  Bahamas  Belize  Belarus  Bermuda  Bolivia  Brunai Darussalam  Burundi  Central African Republic  Comoros  Costa Rica  Cote D’Ivoire  Cuba  Dominican Republic  Djibouti  El Salvador  Fiji  French Polynesia  Gambia  Ghana  Grenada  Guinea  Guinea-Bissau  Guyana  Haiti  Honduras  Kiribati  Lesotho  Liberia  Macedonia  Maldives  Mauritius | Morocco  Myanmar  Namibia  Netherlands Antilles  New Caledonia  Nicaragua  Nigeria  Occupied Palestine territory  Panama  Paraguay  Rwanda  St. Lucia  St. Kitts & Nevis  St. Vincent & the Grenadines  Samoa  Senegal  Sao Tome & Principe  Sudan (former)  Suriname  Swaziland  Syrian Arab Republic  Tajikistan  Timor-Leste  Togo  Turkmenistan  Uganda  United Arab Emirates  United Republic of Tanzania  Uzbekistan  Vietnam  Yemen  Yugoslavia  Zimbabwe | American Samoa  BVI (British Virgin Islands)  Bahrain  Bhutan  Cook Islands  Greenland  Hong Kong  Marshall Islands  Micronesia  Nauru  Papua New Guinea  Singapore  Taiwan  Tokelau  Tonga |

^1^Hand-searching of countries not matched in initial merge due to name differences between databases (different spelling/ phrasing, or country name changes over time updated differently in FAO vs. GDD databases) led to additionally matching of the following countries:

Luxembourg, Czechoslovakia, Iran, Korea, Kyrgyzstan, Lao PDR, Libya, (Republic of) Moldova, Serbia, Montenegro

**Supplementary Figure 1.** GDD means vs. mean differences for age-sex pairs, 1980-2009
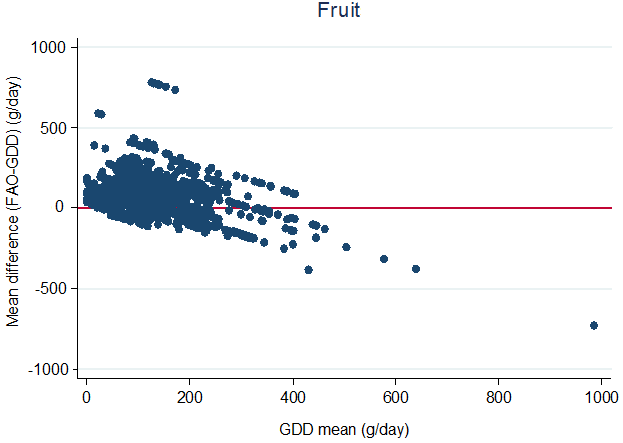


.
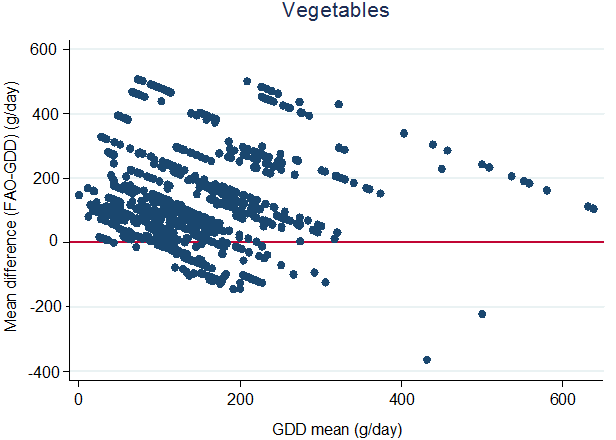


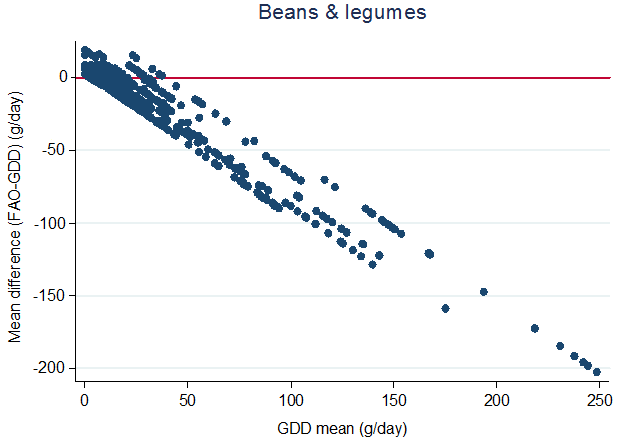


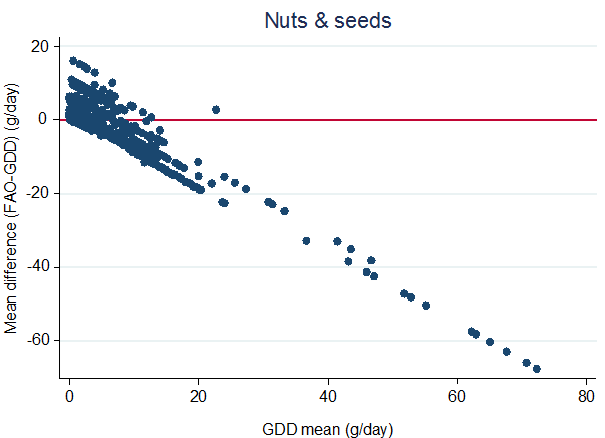


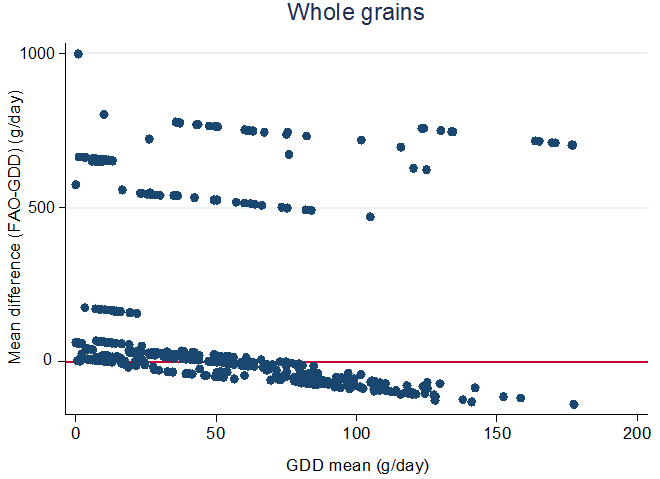


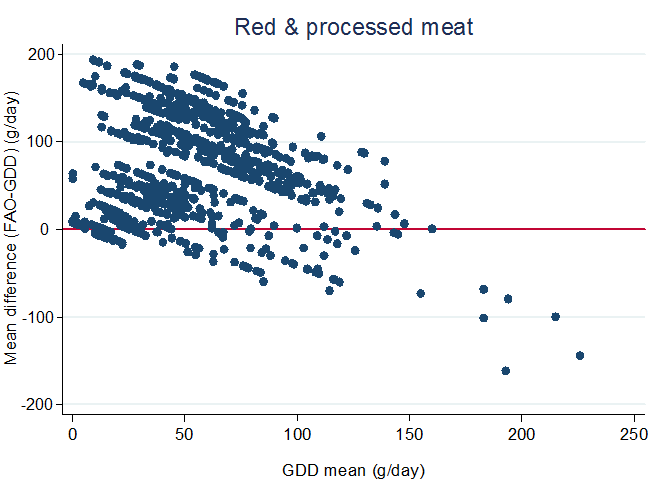


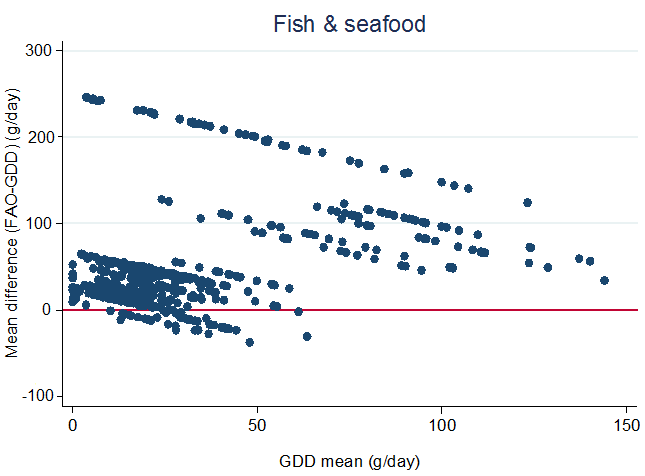


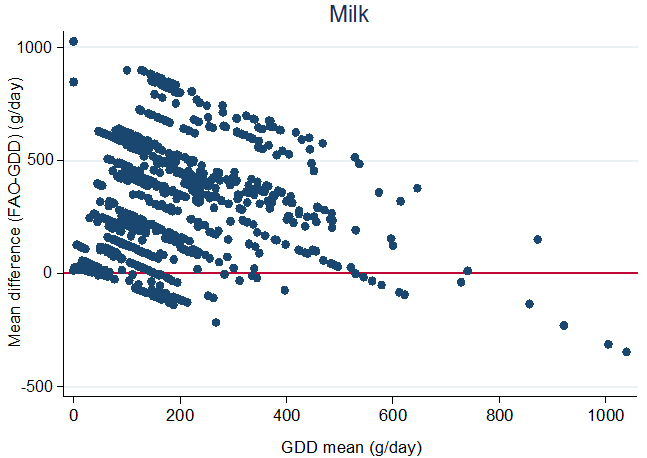


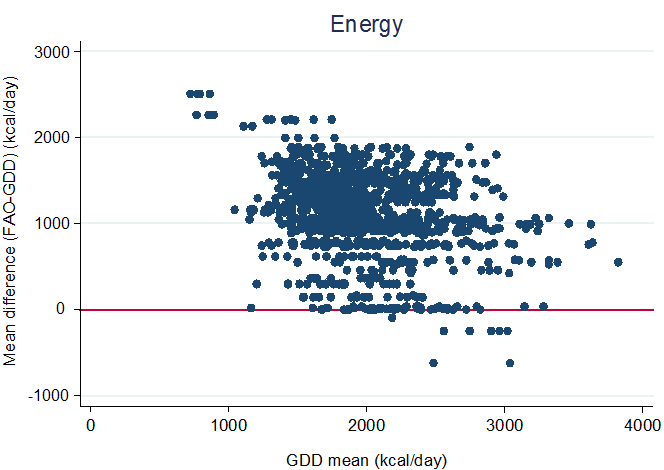


**Supplementary Table 4.** Relation between FAO food availability estimates and other GDD variables as predictors of GDD mean dietary intakes in the full age-sex dataset

| **VARIABLE KEY**  fao_g_day: FAO estimate (g/day), continuous  SEX  sex_d1: Sex: 1=females; 0=males  AGE  *age 20-34y is reference*  age_d1: Age: 35-49y=1; all other ages=0  age_d2: Age: 50-69y=1; all other ages=0  age_d3: Age: ≥70y=1; all other ages=0  REGION  *Asia (South-east/Asia Pacific) is reference*  r_west: North America, Australia & New Zealand, Western Europe=1, all other regions=0 r_afr: Sub-Saharan Africa, North Africa & Middle East=1, all other regions=0 r_centas: Central Asia & Eastern/Central Europe=1, all other regions=0 r_lat= Latin America, Caribbean, Oceania=1, all other regions=0  ASSESSMENT  *Multiple dietary recalls is reference*  diet_d1: Food frequency questionnaire=1, all other assessment methods=0  diet_d2: One recall=1, all other assessment methods=0  REPRESENTATIVENESS  *National representativeness is reference*  repp_d1: Regional representativeness=1, all other representativeness=0  repp_d2: Urban/local/cohort=1, all other representativeness=0  YEAR  *<2000 is reference*  year_d1: 2000-2004=1, all other survey years=0  year_d2: ≥2005=1, all other survey years=0  _cons: constant term  **NOTE**: All regressions are inverse-variance weighted | SEX (Interaction term)  se_d1= fao_g_day*sex_d1  AGE (Interaction terms)  ag_d1=fao_g_day*age_d1  ag_d2=fao_g_day*age_d2  ag_d3=fao_g_day*age_d3  ASSESSMENT (Interaction terms)  die_d1=fao_g_day*diet_d1  die_d2=fao_g_day*diet_d2  REPRESENTATIVENESS (Interaction terms)  rep_d1=fao_g_day*repp_d1  rep_d2=fao_g_day*repp_d2  YEAR (Interaction terms)  yr_d1=fao_g_day*year_d1  yr_d2=fao_g_day*year_d2 |
| --- | --- |

| Fruit  meanGDD \| Coef. Std. Err. t P>\|t\| [95% Conf. Interval]  ------------------+----------------------------------------------------------------  fao_g_day \| .4984225 .1299492 3.84 0.000 .2430734 .7537716  sex_d1 \| -6.175969 13.67831 -0.45 0.652 -33.05374 20.7018  age_d1 \| 46.35649 17.94569 2.58 0.010 11.09336 81.61962  age_d2 \| 37.66917 19.8614 1.90 0.058 -1.358314 76.69665  age_d3 \| 66.29357 28.71507 2.31 0.021 9.868699 122.7184  r_afr \| 27.68538 15.79688 1.75 0.080 -3.355366 58.72613  r_centas \| 35.21095 14.03499 2.51 0.012 7.632299 62.7896  r_west \| 53.31515 15.00388 3.55 0.000 23.83264 82.79766  r_lat \| 179.8578 16.46323 10.92 0.000 147.5076 212.2079  diet_d1 \| 69.90033 39.53301 1.77 0.078 -7.781707 147.5824  diet_d2 \| 114.9376 38.95934 2.95 0.003 38.38278 191.4924  repp_d1 \| 19.29469 17.96543 1.07 0.283 -16.00724 54.59662  repp_d2 \| -54.42948 31.63724 -1.72 0.086 -116.5964 7.73745  year_d1 \| 146.6367 20.2727 7.23 0.000 106.801 186.4723  year_d2 \| 76.97781 23.65311 3.25 0.001 30.49964 123.456  se_d1 \| .1029871 .055836 1.84 0.066 -.0067302 .2127045  ag_d1 \| -.1178928 .0735668 -1.60 0.110 -.262451 .0266654  ag_d2 \| -.0369042 .0805709 -0.46 0.647 -.1952254 .1214171  ag_d3 \| -.2165131 .1056868 -2.05 0.041 -.4241868 -.0088394  die_d1 \| .0358371 .1236753 0.29 0.772 -.2071839 .2788581  die_d2 \| -.2449974 .1277719 -1.92 0.056 -.4960682 .0060734  rep_d1 \| -.2524877 .0859573 -2.94 0.003 -.4213931 -.0835824  rep_d2 \| .3202612 .1058113 3.03 0.003 .1123429 .5281795  yr_d1 \| -.5247521 .0791308 -6.63 0.000 -.6802435 -.3692607  yr_d2 \| -.3128614 .0874986 -3.58 0.000 -.4847955 -.1409273  _cons \| -89.15996 38.3183 -2.33 0.020 -164.4551 -13.8648 | Vegetables  meanGDD \| Coef. Std. Err. t P>\|t\| [95% Conf. Interval]  ------------------+----------------------------------------------------------------  fao_g_day \| .4376261 .0622122 7.03 0.000 .3153743 .559878  sex_d1 \| .2841664 5.619262 0.05 0.960 -10.75812 11.32646  age_d1 \| -3.343436 7.018897 -0.48 0.634 -17.13612 10.44925  age_d2 \| -9.488545 7.704655 -1.23 0.219 -24.6288 5.651708  age_d3 \| -26.04938 9.882384 -2.64 0.009 -45.46905 -6.629721  r_afr \| 53.03049 7.591579 6.99 0.000 38.11244 67.94854  r_centas \| 80.65348 9.438558 8.55 0.000 62.10597 99.20099  r_west \| 14.9764 7.785264 1.92 0.055 -.3222616 30.27505  r_lat \| -16.47587 7.051245 -2.34 0.020 -30.33213 -2.619621  diet_d1 \| -32.30965 14.88335 -2.17 0.030 -61.55659 -3.062698  diet_d2 \| 31.97374 19.07914 1.68 0.094 -5.518277 69.46577  repp_d1 \| 28.55416 11.35723 2.51 0.012 6.236312 50.872  repp_d2 \| -70.11065 29.45836 -2.38 0.018 -127.9987 -12.22265  year_d1 \| 138.451 16.17414 8.56 0.000 106.6675 170.2344  year_d2 \| 52.81051 13.05904 4.04 0.000 27.14846 78.47256  se_d1 \| -.0054847 .0300877 -0.18 0.855 -.0646094 .0536401  ag_d1 \| .0244976 .0382495 0.64 0.522 -.0506657 .0996608  ag_d2 \| .068311 .0424258 1.61 0.108 -.0150589 .151681  ag_d3 \| .1613402 .0531094 3.04 0.003 .0569761 .2657043  die_d1 \| .039058 .0643634 0.61 0.544 -.0874212 .1655372  die_d2 \| -.0868289 .0624297 -1.39 0.165 -.2095083 .0358504  rep_d1 \| -.0874347 .0591003 -1.48 0.140 -.2035714 .028702  rep_d2 \| .3242128 .0865541 3.75 0.000 .1541272 .4942984  yr_d1 \| -.7377907 .0705713 -10.45 0.000 -.8764689 -.5991125  yr_d2 \| -.3170913 .0509377 -6.23 0.000 -.4171879 -.2169947  _cons \| -16.37954 14.93725 -1.10 0.273 -45.73241 12.97333 |
| --- | --- |
| Beans & legumes  meanGDD \| Coef. Std. Err. t P>\|t\| [95% Conf. Interval]  ------------------+----------------------------------------------------------------  fao_g_day \| 4.3599 .7676232 5.68 0.000 2.849815 5.869986  sex_d1 \| -2.103365 3.459448 -0.61 0.544 -8.908869 4.702139  age_d1 \| -1.986221 4.165179 -0.48 0.634 -10.18006 6.207615  age_d2 \| -6.796932 4.038683 -1.68 0.093 -14.74192 1.148057  age_d3 \| -1.210532 6.678633 -0.18 0.856 -14.34889 11.92783  r_afr \| .1197936 6.497307 0.02 0.985 -12.66186 12.90144  r_centas \| 7.027163 6.857187 1.02 0.306 -6.462451 20.51678  r_west \| -19.77876 3.801562 -5.20 0.000 -27.25728 -12.30024  r_lat \| 15.69585 4.997653 3.14 0.002 5.864354 25.52735  diet_d1 \| 37.21178 8.752115 4.25 0.000 19.99442 54.42914  diet_d2 \| 53.75231 7.790922 6.90 0.000 38.42583 69.0788  repp_d1 \| -77.01321 24.08505 -3.20 0.002 -124.3939 -29.63255  repp_d2 \| 51.09382 9.403018 5.43 0.000 32.59598 69.59165  year_d1 \| 2.867014 8.249649 0.35 0.728 -13.36188 19.09591  year_d2 \| -11.85984 7.169942 -1.65 0.099 -25.96471 2.245038  se_d1 \| -.8519872 .1745984 -4.88 0.000 -1.195461 -.5085132  ag_d1 \| -.2320151 .1602536 -1.45 0.149 -.5472696 .0832394  ag_d2 \| .0277537 .1634897 0.17 0.865 -.293867 .3493744  ag_d3 \| -.366626 .2209367 -1.66 0.098 -.8012578 .0680059  die_d1 \| -3.495645 .3335261 -10.48 0.000 -4.151765 -2.839525  die_d2 \| -5.89986 .7491609 -7.88 0.000 -7.373626 -4.426093  rep_d1 \| 10.04289 2.294995 4.38 0.000 5.528124 14.55766  rep_d2 \| .190153 .6961528 0.27 0.785 -1.179335 1.559641  yr_d1 \| -.9771645 .7693243 -1.27 0.205 -2.490597 .5362678  yr_d2 \| .7196734 .7284746 0.99 0.324 -.7133984 2.152745  _cons \| 12.73651 9.17638 1.39 0.166 -5.31548 30.78849 | Nuts & seeds  meanGDD \| Coef. Std. Err. t P>\|t\| [95% Conf. Interval]  ------------------+----------------------------------------------------------------  fao_g_day \| .6837683 .402161 1.70 0.090 -.1069001 1.474437  sex_d1 \| .1558803 .4586473 0.34 0.734 -.7458432 1.057604  age_d1 \| .334266 .8287602 0.40 0.687 -1.295118 1.96365  age_d2 \| .9914701 .7498572 1.32 0.187 -.4827865 2.465727  age_d3 \| 1.03538 .6071978 1.71 0.089 -.1584011 2.229161  r_afr \| 6.332018 2.263256 2.80 0.005 1.882344 10.78169  r_centas \| -2.132162 1.503209 -1.42 0.157 -5.087545 .8232217  r_west \| 3.0869 .6117132 5.05 0.000 1.884241 4.289558  r_lat \| -3.169034 1.349906 -2.35 0.019 -5.823016 -.5150514  diet_d1 \| .7117861 1.266954 0.56 0.575 -1.779109 3.202681  diet_d2 \| 1.000487 .9987145 1.00 0.317 -.9630349 2.96401  repp_d1 \| 5.925289 2.242749 2.64 0.009 1.515933 10.33464  repp_d2 \| 11.24009 3.888868 2.89 0.004 3.594382 18.8858  year_d1 \| -.867125 1.069866 -0.81 0.418 -2.970534 1.236284  year_d2 \| -.2894405 1.13611 -0.25 0.799 -2.52309 1.944209  se_d1 \| -.0307944 .1614358 -0.19 0.849 -.3481852 .2865964  ag_d1 \| -.071764 .3157004 -0.23 0.820 -.6924467 .5489186  ag_d2 \| -.3894976 .2756731 -1.41 0.158 -.9314845 .1524894  ag_d3 \| -.6446181 .2025199 -3.18 0.002 -1.042782 -.2464538  die_d1 \| 1.176843 .3197257 3.68 0.000 .5482464 1.80544  die_d2 \| .2486091 .2419268 1.03 0.305 -.2270311 .7242493  rep_d1 \| -2.058173 .4049142 -5.08 0.000 -2.854255 -1.262092  rep_d2 \| -3.57617 1.032104 -3.46 0.001 -5.605338 -1.547003  yr_d1 \| .84821 .3157973 2.69 0.008 .2273368 1.469083  yr_d2 \| 1.031832 .3279426 3.15 0.002 .3870808 1.676584  _cons \| -2.058531 1.309232 -1.57 0.117 -4.632547 .5154852 |
| Whole grains  meanGDD \| Coef. Std. Err. t P>\|t\| [95% Conf. Interval]  ------------------+----------------------------------------------------------------  fao_g_day \| -.4804294 .0666868 -7.20 0.000 -.6122487 -.3486102  sex_d1 \| -9.186489 11.91914 -0.77 0.442 -32.74695 14.37398  age_d1 \| -32.4125 15.63207 -2.07 0.040 -63.3123 -1.512703  age_d2 \| -13.01144 16.50669 -0.79 0.432 -45.64009 19.61721  age_d3 \| -24.24194 18.6895 -1.30 0.197 -61.18533 12.70145  r_afr \| -5.381509 20.58806 -0.26 0.794 -46.07777 35.31475  r_centas \| 11.01349 15.22107 0.72 0.471 -19.07388 41.10087  r_west \| 41.13409 6.611792 6.22 0.000 28.06461 54.20356  r_lat \| 46.28604 8.070155 5.74 0.000 30.33383 62.23826  diet_d1 \| 54.33655 49.6269 1.09 0.275 -43.76055 152.4337  diet_d2 \| 2.682087 14.9619 0.18 0.858 -26.89299 32.25717  repp_d1 \| 0 (omitted)  repp_d2 \| -188.9532 53.82716 -3.51 0.001 -295.3529 -82.55345  year_d1 \| -151.1165 22.51769 -6.71 0.000 -195.6271 -106.606  year_d2 \| -81.44249 23.90745 -3.41 0.001 -128.7002 -34.18482  se_d1 \| .0140861 .0460027 0.31 0.760 -.0768472 .1050194  ag_d1 \| .1181112 .0625106 1.89 0.061 -.0054531 .2416754  ag_d2 \| .0479474 .0662198 0.72 0.470 -.0829487 .1788435  ag_d3 \| .0949299 .0750877 1.26 0.208 -.0534954 .2433551  die_d1 \| -.0277093 .2294468 -0.12 0.904 -.4812549 .4258364  die_d2 \| -.0102356 .0614237 -0.17 0.868 -.1316513 .1111802  rep_d1 \| -.1719122 .21462 -0.80 0.424 -.5961499 .2523254  rep_d2 \| .4397571 .1354301 3.25 0.001 .1720535 .7074608  yr_d1 \| .5021709 .0822869 6.10 0.000 .3395149 .6648268  yr_d2 \| .2225086 .0842733 2.64 0.009 .0559262 .3890909  _cons \| 159.0098 16.14373 9.85 0.000 127.0986 190.9209 | Red & processed meat  meanGDD \| Coef. Std. Err. t P>\|t\| [95% Conf. Interval]  -------------+----------------------------------------------------------------  fao_g_day \| -.0836427 .1437387 -0.58 0.561 -.3664383 .1991529  sex_d1 \| -32.0632 8.476203 -3.78 0.000 -48.73952 -15.38688  age_d1 \| 7.25833 10.61134 0.68 0.494 -13.61872 28.13538  age_d2 \| 20.4971 10.27596 1.99 0.047 .2798735 40.71432  age_d3 \| -17.18467 14.74835 -1.17 0.245 -46.20099 11.83165  r_afr \| -21.0898 11.55742 -1.82 0.069 -43.8282 1.648606  r_centas \| 31.78544 5.607552 5.67 0.000 20.75298 42.8179  r_west \| -10.49392 6.992126 -1.50 0.134 -24.25043 3.262589  r_lat \| 37.48714 9.873321 3.80 0.000 18.06209 56.91219  diet_d1 \| -45.6392 17.7425 -2.57 0.011 -80.5463 -10.73209  diet_d2 \| -25.23196 16.84535 -1.50 0.135 -58.37397 7.91006  repp_d1 \| -23.95009 25.00276 -0.96 0.339 -73.14123 25.24105  repp_d2 \| -19.61188 16.47722 -1.19 0.235 -52.02963 12.80586  year_d1 \| -72.29885 13.99784 -5.16 0.000 -99.8386 -44.7591  year_d2 \| -29.72288 11.61773 -2.56 0.011 -52.57993 -6.865824  se_d1 \| -.016937 .0602398 -0.28 0.779 -.1354545 .1015804  ag_d1 \| -.0166256 .0729697 -0.23 0.820 -.1601883 .126937  ag_d2 \| -.1268393 .0763714 -1.66 0.098 -.2770945 .023416  ag_d3 \| .0588246 .1062415 0.55 0.580 -.1501981 .2678472  die_d1 \| .3116004 .1162916 2.68 0.008 .082805 .5403958  die_d2 \| .2223979 .1151951 1.93 0.054 -.0042401 .4490359  rep_d1 \| -.1511815 .160554 -0.94 0.347 -.46706 .164697  rep_d2 \| .1023142 .1464407 0.70 0.485 -.1857973 .3904258  yr_d1 \| .2760618 .0915806 3.01 0.003 .0958836 .4562401  yr_d2 \| .0278008 .0975331 0.29 0.776 -.1640886 .2196902  _cons \| 116.4229 19.477 5.98 0.000 78.1033 154.7425 |
| Fish & seafood  meanGDD \| Coef. Std. Err. t P>\|t\| [95% Conf. Interval]  -------------+----------------------------------------------------------------  fao_g_day \| .1966371 .0412034 4.77 0.000 .1155581 .2777161  sex_d1 \| -1.702568 1.535891 -1.11 0.269 -4.724851 1.319715  age_d1 \| -.590879 1.83904 -0.32 0.748 -4.209691 3.027933  age_d2 \| -1.082879 1.979751 -0.55 0.585 -4.978577 2.81282  age_d3 \| -1.024278 2.548272 -0.40 0.688 -6.038696 3.990141  r_afr \| -27.92245 7.422066 -3.76 0.000 -42.52738 -13.31751  r_centas \| -33.43327 2.258717 -14.80 0.000 -37.87791 -28.98863  r_west \| -33.37758 1.36646 -24.43 0.000 -36.06647 -30.6887  r_lat \| -17.11908 5.638784 -3.04 0.003 -28.21492 -6.023235  diet_d1 \| -11.45266 8.384945 -1.37 0.173 -27.95232 5.047002  diet_d2 \| 7.406805 3.320903 2.23 0.026 .872024 13.94159  repp_d1 \| -2.405773 13.27546 -0.18 0.856 -28.52886 23.71731  repp_d2 \| 13.21174 16.66811 0.79 0.429 -19.5873 46.01078  year_d1 \| 3.799165 1.775589 2.14 0.033 .3052096 7.29312  year_d2 \| -.8811493 3.499983 -0.25 0.801 -7.768319 6.00602  se_d1 \| -.0959771 .012259 -7.83 0.000 -.1200999 -.0718543  ag_d1 \| .0276511 .0143901 1.92 0.056 -.0006654 .0559676  ag_d2 \| .0425233 .0166446 2.55 0.011 .0097705 .0752761  ag_d3 \| .0352676 .0189184 1.86 0.063 -.0019595 .0724946  die_d1 \| .3439407 .0756595 4.55 0.000 .19506 .4928214  die_d2 \| .1110605 .0417772 2.66 0.008 .0288525 .1932685  rep_d1 \| -.0490224 .521535 -0.09 0.925 -1.075285 .9772399  rep_d2 \| -.2033125 .1030897 -1.97 0.049 -.4061696 -.0004554  yr_d1 \| -.1787056 .0136349 -13.11 0.000 -.205536 -.1518752  yr_d2 \| -.1437057 .0264499 -5.43 0.000 -.1957532 -.0916583  _cons \| 40.30161 3.131682 12.87 0.000 34.13918 46.46405 | Milk  meanGDD \| Coef. Std. Err. t P>\|t\| [95% Conf. Interval]  -------------+----------------------------------------------------------------  fao_g_day \| .2170392 .0721941 3.01 0.003 .0751867 .3588916  sex_d1 \| 27.57254 30.82981 0.89 0.372 -33.00425 88.14934  age_d1 \| -8.639638 38.35349 -0.23 0.822 -83.99954 66.72027  age_d2 \| 20.8613 39.21211 0.53 0.595 -56.1857 97.90829  age_d3 \| -70.3732 58.9769 -1.19 0.233 -186.2556 45.50918  r_afr \| 74.33919 42.76827 1.74 0.083 -9.695218 158.3736  r_centas \| 87.67397 31.50943 2.78 0.006 25.76181 149.5861  r_west \| -2.975992 35.61374 -0.08 0.933 -72.95263 67.00064  r_lat \| 31.45299 38.68813 0.81 0.417 -44.56443 107.4704  diet_d1 \| -139.4666 58.7585 -2.37 0.018 -254.9198 -24.01333  diet_d2 \| -26.76077 51.32542 -0.52 0.602 -127.6089 74.08739  repp_d1 \| 109.2505 97.04905 1.13 0.261 -81.43902 299.94  repp_d2 \| -22.74119 64.29477 -0.35 0.724 -149.0725 103.5902  year_d1 \| 118.4146 40.64193 2.91 0.004 38.55816 198.271  year_d2 \| -66.41 48.82981 -1.36 0.174 -162.3546 29.53459  se_d1 \| -.0756369 .0486758 -1.55 0.121 -.1712789 .020005  ag_d1 \| -.0090247 .0603851 -0.15 0.881 -.1276741 .1096246  ag_d2 \| -.066775 .0625233 -1.07 0.286 -.1896256 .0560755  ag_d3 \| .0303218 .1002592 0.30 0.762 -.1666751 .2273188  die_d1 \| .5516109 .0897763 6.14 0.000 .3752115 .7280102  die_d2 \| .203977 .0701703 2.91 0.004 .0661008 .3418531  rep_d1 \| -.6729973 .1980974 -3.40 0.001 -1.062234 -.2837602  rep_d2 \| -.3537478 .179543 -1.97 0.049 -.7065277 -.0009679  yr_d1 \| -.1463272 .0653826 -2.24 0.026 -.2747959 -.0178585  yr_d2 \| .1039497 .0760139 1.37 0.172 -.0454084 .2533077  _cons \| 75.88787 56.32246 1.35 0.178 -34.77886 186.5546 |
| Energy  meanGDD \| Coef. Std. Err. t P>\|t\| [95% Conf. Interval]  -------------+----------------------------------------------------------------  FAO_kcal_day \| .4208566 .100646 4.18 0.000 .2232568 .6184564  sex_d1 \| 518.9823 173.2693 3.00 0.003 178.7999 859.1647  age_d1 \| 252.649 236.8892 1.07 0.287 -212.4393 717.7374  age_d2 \| -125.7351 230.3921 -0.55 0.585 -578.0674 326.5972  age_d3 \| -8.885604 285.9554 -0.03 0.975 -570.3063 552.535  r_afr \| 106.3533 56.70191 1.88 0.061 -4.970474 217.677  r_centas \| 64.89643 40.24352 1.61 0.107 -14.1143 143.9072  r_west \| 1.995552 45.62214 0.04 0.965 -87.5751 91.5662  r_lat \| 78.13113 65.35709 1.20 0.232 -50.18547 206.4477  diet_d1 \| 256.2147 309.3697 0.83 0.408 -351.1756 863.605  diet_d2 \| 363.7628 267.8056 1.36 0.175 -162.0241 889.5497  repp_d1 \| 582.6432 364.925 1.60 0.111 -133.8197 1299.106  repp_d2 \| 1041.105 271.3405 3.84 0.000 508.3776 1573.832  year_d1 \| -129.9532 225.2051 -0.58 0.564 -572.1018 312.1954  year_d2 \| 68.26439 316.6519 0.22 0.829 -553.4232 689.952  se_d1 \| -.297486 .0544234 -5.47 0.000 -.4043363 -.1906357  ag_d1 \| -.0686105 .0745371 -0.92 0.358 -.2149504 .0777294  ag_d2 \| .0477263 .0731589 0.65 0.514 -.0959078 .1913604  ag_d3 \| -.0007465 .0898378 -0.01 0.993 -.1771265 .1756335  die_d1 \| .0260267 .099387 0.26 0.793 -.1691013 .2211548  die_d2 \| -.0883815 .0831061 -1.06 0.288 -.251545 .074782  rep_d1 \| -.2056973 .11903 -1.73 0.084 -.4393907 .0279962  rep_d2 \| -.3964866 .0912707 -4.34 0.000 -.5756799 -.2172934  yr_d1 \| .0266715 .0728683 0.37 0.714 -.1163919 .1697349  yr_d2 \| -.0547619 .0967966 -0.57 0.572 -.2448042 .1352805  _cons \| 846.0345 306.3231 2.76 0.006 244.6256 1447.443 |  |
